# Sex differences in the outcomes of stent implantation in mini-swine model

**DOI:** 10.1371/journal.pone.0192004

**Published:** 2018-01-29

**Authors:** Mie Kunio, Gee Wong, Peter M. Markham, Elazer R. Edelman

**Affiliations:** 1 Institute for Medical Engineering and Science, Massachusetts Institute of Technology, Cambridge, Massachusetts, United States of America; 2 CBSET, Inc., Lexington, Massachusetts, United States of America; 3 Cardiovascular Division, Brigham and Women’s Hospital, Harvard Medical School, Boston, Massachusetts, United States of America; University of California Berkeley, UNITED STATES

## Abstract

Sex-related differences have been noted in cardiovascular anatomy, pathophysiology, and treatment responses, yet we continued to drive evaluation of vascular device development in animal models without consideration of animal sex. We aimed to understand sex-related differences in the vascular responses to stent implantation by analyzing the pooled data of endovascular interventions in 164 Yucatan mini-swine (87 female, 77 male). Bare metal stents (BMS) or drug-eluting stents (DES) were implanted in 212 coronary arteries (63 single BMS implantation, 68 single DES implantation, 33 overlapped BMS implantation, and 48 overlapped DES implantation). Histomorphological parameters were evaluated from vascular specimens at 3–365 days after stent implantation and evaluated values were compared between female and male groups. While neointima formation at all times after implantation was invariant to sex, statistically significant differences between female and male groups were observed in injury, inflammation, adventitial fibrosis, and neointimal fibrin deposition. These differences were observed independently, i.e., for different procedure types and at different follow-up timings. Only subtle temporal sex-related differences were observed in extent and timing of resolution of inflammation and fibrin clearance. These subtle sex-related differences may be increasingly important as interventional devices meld novel materials that erode and innovations in drug delivery. Erodible materials may act differently if inflammation has a different temporal sequence with sex, and drug distribution after balloon or stent delivery might be different if the fibrin clearance speaks to different modes of pharmacokinetics in male and female swine.

## Introduction

Cardiovascular disease (CVD) is the main cause of death in women and men around the world [1]. However, like most diseases, its diagnostic criteria and treatments protocols have been developed from studies dominated by enrollment with male patients. In 2001, the Institute of Medicine declared that: “sex differences should be considered in all health-related research, research should concentrate on sex-differences in diagnosis and treatment, and on the mechanisms and therapies related to sex differences, and obstacles to advance sex-related research in health and illness must be eliminated” [2]. A large amount of research has identified sex differences that may affect the symptoms and therapeutic outcomes of CVD, such as anatomic and physiologic differences. Arterial diameters and wall thickness are thinner in women than in men [3–5], and left atrial and ventricular end-diastolic dimensions are different [6]. The basic pathology of arterial disease is indeed different between men and women. Necrotic lesions are more evident in women, while men demonstrate more calcified, fibrous, and fibrofatty plaques [7, 8]. It can therefore be understood why plaque erosion with acute thrombosis was observed more in women than in men (men: 18.5%, women: 37.4%), and rupture more frequently in men (men: 76%, women: 55%) [9, 10]. The onset of CVD is about 10 years later in women than in men [8, 11].

Concern has been raised that these differences might herald sex-related difference in therapeutic outcomes. Drug therapies for atherosclerosis and CVD have made great advances, but women have not derived as much benefit as men. Women tend to experience more drug-related adverse events than men, perhaps from differences in body mass, volume of drug distribution, liver metabolism, and/or kidney function [8]. Some clinical trials for drugs, including those for aspirin, the most prevalent drug used for CVD, have addressed this disparity [11]. Although a meta-analysis of primary prevention studies did not suggest sex differences in the response to aspirin, differences were evident in secondary studies, which showed that a first stroke, but not a first heart attack, is prevented with a low dose of aspirin in women, and that the reverse is true in men (i.e., a first heart attack but not a first stroke is prevented) [11]. The cause of the difference in the effect of aspirin has not yet been delineated [12], but this difference has been attributed to differences in platelet biology, including especially higher baseline platelet reactivity in response to agonists in women than in men has been reported [13]. The efficacy of statins also demonstrates sex-related differences. Secondary prevention trials show that statins seem to work equally in women and men with established CVD; however, in the recent large primary prevention trial, more robust reduction in cardiac event with statin therapy was observed in women than in men without an evident CVD risk [11].

In addition, sex-related differences in the outcomes of stent implantation have been studied [14–43]; however, the results are controversial for bare metal stent (BMS) and drug-eluting stent (DES) implantation. For example in BMS implantation, Mehilli et al. demonstrated higher rates of death, non-fatal myocardial infarction, and urgent target vessel revascularization in women than in men within 30 days after the implantation, and lower rates of clinical restenosis rate and target vessel revascularization in women than in men at 1-year follow-up [42]. On the other hand, Lansky et al. demonstrated no sex-related difference between women and men at any follow-up timing [38]. Little research focuses on sex-related differences in the outcomes of DES implantation. One study demonstrated that no sex-related differences were observed in the outcomes of Sirolimus-eluting stent implantation after adjusting for the sex-related differences in confounding variables [33, 36]. Another study showed that women undergoing Zotarolimus-eluting stent implantation tend to have higher in-stent and in-segment percent diameter restenosis and lower rates of revascularization, but have similar rates of death and myocardial infarction compared to men [44]. The difference between these two studies were attributed to drugs differences: Sirolimus might induce more thrombotic events due to inflammation response, while Zotarolimus reduces thrombotic events [25], or sex-related difference in platelet biology. However, in all of these clinical trials, the percentages of women were about 20–30% and women tended to be older and have more risks of CVD than men. This difference may induce the ambiguous results in clinical trials and prevent us from understanding the purely sex-related differences in the outcomes of stent implantation.

The National Institutes of Health now use consideration of sex as a biological variable as a dominant criterion in evaluation of research funding proposals and yet sex has rarely been evaluated in animal model systems. These systems are an essential bedrock of research into vascular biology and in the determination of the safety/efficacy of endovascular interventions. Animal systems are ideal for testing specific hypotheses that drive vascular disease and much of what we know of vascular biology is derived from animal work. Endovascular therapies require evaluation in animal systems before clinical evaluation can commence. Yet, few if any studies are concerned with the choice of sex in preclinical studies. It is for this reason that we pooled data from extensive experience in preclinical studies to determine if sex-related differences could be identified in the vascular responses to the stent implantation.

## Materials and methods

### Animal study population

All animal studies were performed at GLP AAALAC accredited animal facility (CBSET, Inc., Lexington, MA) and adhered to the Guide for the Care and Use of Laboratory Animals under an Institutional Animal Care and Use committee-approved protocol. We pooled data from 164 Yucatan mini-swine (4-month old). Among all mini-swine, 87 (53%) were nulliparous female and 77 (47%) were castrated male. BMS or DES was implanted in the three main coronary arteries with standard procedure. The procedure is described in S1 Appendix. Within 212 stented vessels in total, 63 (29.7%) were single-usage of BMS, 68 (32.1%) were single-usage of DES, 33 (15.6%) were overlapped-usage of BMS, and 48 (22.6%) were overlapped-usage of DES (Table 1). The baseline diameter and post-implant diameter are summarized in Table 2.

**Table 1:** Summary of available animal data.

Time point	All mini-swine				Male mini-swine				Female mini-swine			
	Single		Overlap		Single		Overlap		Single		Overlap	
	BMS	DES	BMS	DES	BMS	DES	BMS	DES	BMS	DES	BMS	DES
Day 3	8	18	8	8	2	8	2	3	6	10	6	5
Day 30	18	27	8	20	12	15	4	9	6	12	4	11
Day 90	20	23	9	20	12	10	6	11	8	13	3	9
Day 180	9	0	8	0	4	0	5	0	5	0	3	0
Day 365	8	0	0	0	3	0	0	0	5	0	0	0
Totals	63	68	33	48	33	33	17	23	30	35	16	25

**Table 2:** Summary of baseline diameter and post-implant diameter.

**Baseline diameter**				
**Procedure type**	**Follow-up day**	**Male [mm]**	**Female [mm]**	***p*-value**
BMS single	Day 3	2.73	2.86 ± 0.24	0.498
	Day 30	2.78 ± 0.15	2.69 ± 0.11	0.224
	Day 90	2.71 ± 0.15	2.73 ± 0.14	0.729
	Day 180	2.75 ± 0.09	2.70 ± 0.13	0.402
	Day 365	2.86 ± 0.17	2.83 ± 0.10	0.717
BMS overlap	Day 3	2.75	2.88 ± 0.17	0.362
	Day 30	2.71 ± 0.08	2.70 ± 0.07	0.892
	Day 90	2.77 ± 0.05	2.90 ± 0.01	0.001*
	Day 180	2.65 ± 0.08	2.85 ± 0.02	0.006*
DES single	Day 3	2.79 ± 0.11	2.74 ± 0.15	0.354
	Day 30	2.79 ± 0.17	2.74 ± 0.17	0.509
	Day 90	2.67 ± 0.14	2.74 ± 0.15	0.321
	Day 180	2.73 ± 0.12	2.78 ± 0.13	0.374
	Day 365	2.89 ± 0.13	2.77 ± 0.20	0.292
DES overlap	Day 3	2.77 ± 0.19	2.97 ± 0.09	0.086
	Day 30	2.77 ± 0.11	2.78 ± 0.15	0.924
	Day 90	2.79 ± 0.09	2.80 ± 0.16	0.895
	Day 180	2.88 ± 0.11	2.76 ± 0.15	0.091
**Post-implant diameter**				
**Procedure type**	**Follow-up day**	**Male [mm]**	**Female [mm]**	***p*-value**
BMS single	Day 3	2.97	3.09 ± 0.20	0.454
	Day 30	2.90 ± 0.23	2.83 ± 0.26	0.588
	Day 90	2.90 ± 0.18	2.95 ± 0.21	0.629
	Day 180	2.89 ± 0.13	2.87 ± 0.18	0.782
	Day 365	3.02 ± 0.29	3.03 ± 0.10	0.955
BMS overlap	Day 3	2.84	3.08 ± 0.24	0.229
	Day 30	2.94 ± 0.14	2.97 ± 0.10	0.773
	Day 90	3.01 ± 0.12	3.10 ± 0.07	0.207
	Day 180	2.93 ± 0.08	3.08 ± 0.03	0.021*
DES single	Day 3	2.94 ± 0.27	2.91 ± 0.17	0.747
	Day 30	2.92 ± 0.20	2.95 ± 0.27	0.806
	Day 90	2.91 ± 0.19	2.88 ± 0.19	0.765
	Day 180	3.03 ± 0.18	3.03 ± 0.13	0.987
	Day 365	3.09 ± 0.15	2.93 ± 0.20	0.222
DES overlap	Day 3	2.88 ± 0.09	3.13 ± 0.08	0.007*
	Day 30	3.03 ± 0.20	3.04 ± 0.20	0.967
	Day 90	3.07 ± 0.14	3.02 ± 0.19	0.538
	Day 180	3.17 ± 0.15	3.04 ± 0.25	0.260

All the values are evaluated as average ± standard deviation. If the number of data is smaller than 3, only average is evaluated.

The mark ‘*’ shows at which procedure type and follow-up timing a statistically significant difference was observed between male and female groups.

Histological specimens were taken from proximal, middle, and distal regions of each stent-implanted vessel at different times (3, 30, 90, 180, and 365 days) after stent implantation. From each histology specimen, neointima area (*A_N_*) and lumen area (*A_L_*) were measured, and luminal occlusion (*O_L_*) was calculated as follows: *O_L_* = *A_N_*/(*A_N_*+*A_L_*). Histomorphological parameters, i.e., injury score, inflammation score, adventitial fibrosis score, neointimal fibrin score, neointimal maturation score, and endothelialization score, were also evaluated from each specimen (Table 3). All the evaluations were performed at CBSET, Inc., and its process is described in S2 Appendix.

**Table 3:** Scoring scheme for histomorphological parameters.

	0	1	2	3	4
**Injury**	No injury; internal elastic lamina intact	Disruption of internal elastic lamina	Disruption of tunica media	Disruption of external elastic lamina/tunica adventitia	N/A
**Inflammation**	No cells present	< 20 cells associated with stent strut	> 20 cells associated with stent strut; with or without tissue effacement; little to no impact on tissue function	> 20 cells associated with stent strut; adjacent vascular tissue effacement; adverse impact on tissue function	N/A
**Neointimal fibrin**	Absent	Infrequent spotting of fibrin	Heavier deposition of fibrin	Heavy deposition of fibrin spanning between struts	N/A
**Neointimal maturation**	Absent	Immature, predominantly fibrino-vascular tissue	Transitional, predominantly organizing smooth muscle	Mature, generalized organized smooth muscle	N/A
**Adventitial fibrosis**	Absent	Minimal presence of fibrous tissue	Notable fibrous tissue in 25–50% of artery circumference	Notable fibrous tissue in > 50% of artery circumference	N/A
**Endothelialization**	Absent	< 25%	25–75%	> 75%	100%, confluence

Injury and inflammation scores were assessed on a per strut basis, and the average of these strut scores represent the aggregate planar score. All other parameters were planar scores.

### Statistical analysis

After dividing the data into female and male groups, the data was subcategorized based on the stent type (BMS or DES), the single- or overlapped-usage, and the follow-up timing. We could not subcategorize the data based on the artery and its region (proximal, middle, or distal) because of the limited sample size. Average and standard deviation (SD) were calculated for all evaluated values in each subcategorized group. The statistical significance between female and male groups was evaluated in each subcategorized group by applying Student’s *t*-test.

## Results

### Neointima area and luminal occlusion

**Fig 1:**
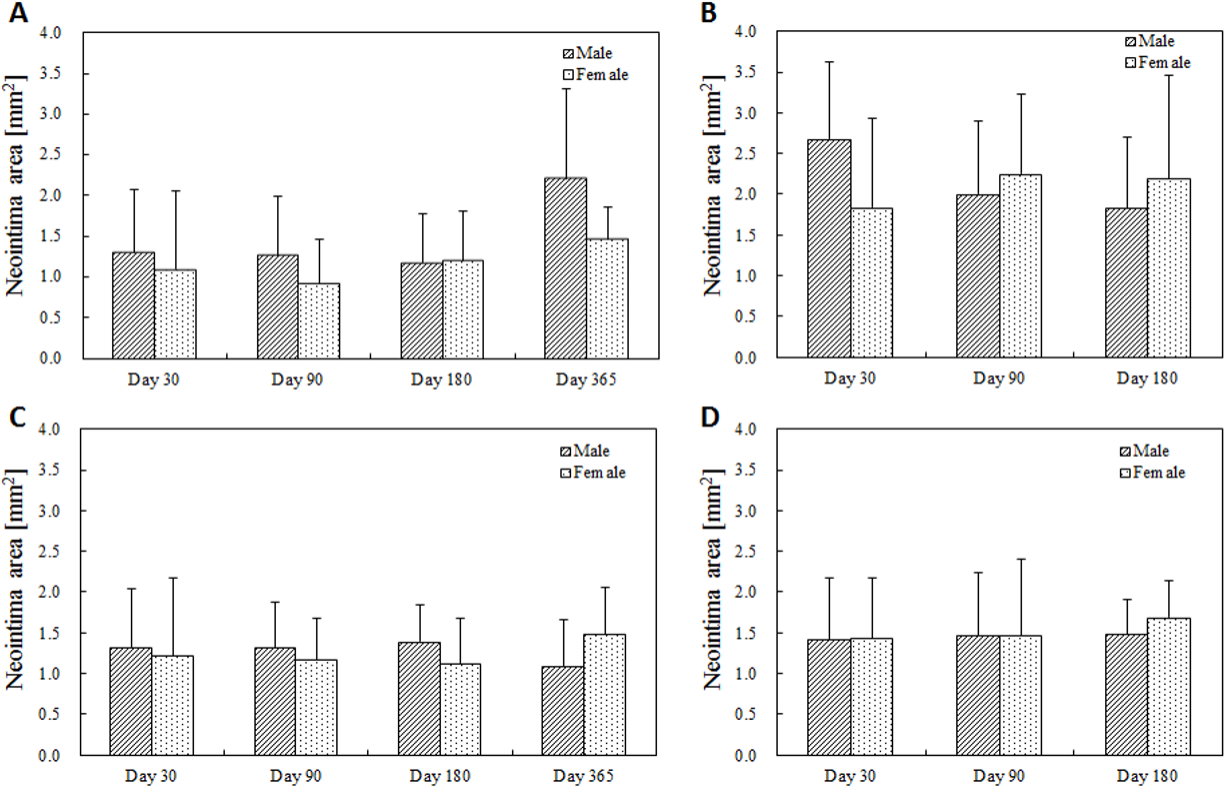
Neointima area at each follow-up timing. (A) Single BMS implantation, (B) Overlapped BMS implantation, (C) Single DES implantation, (D) Overlapped DES implantation. No significant differences between female and male groups were observed at any following timings.

**Fig 2:**
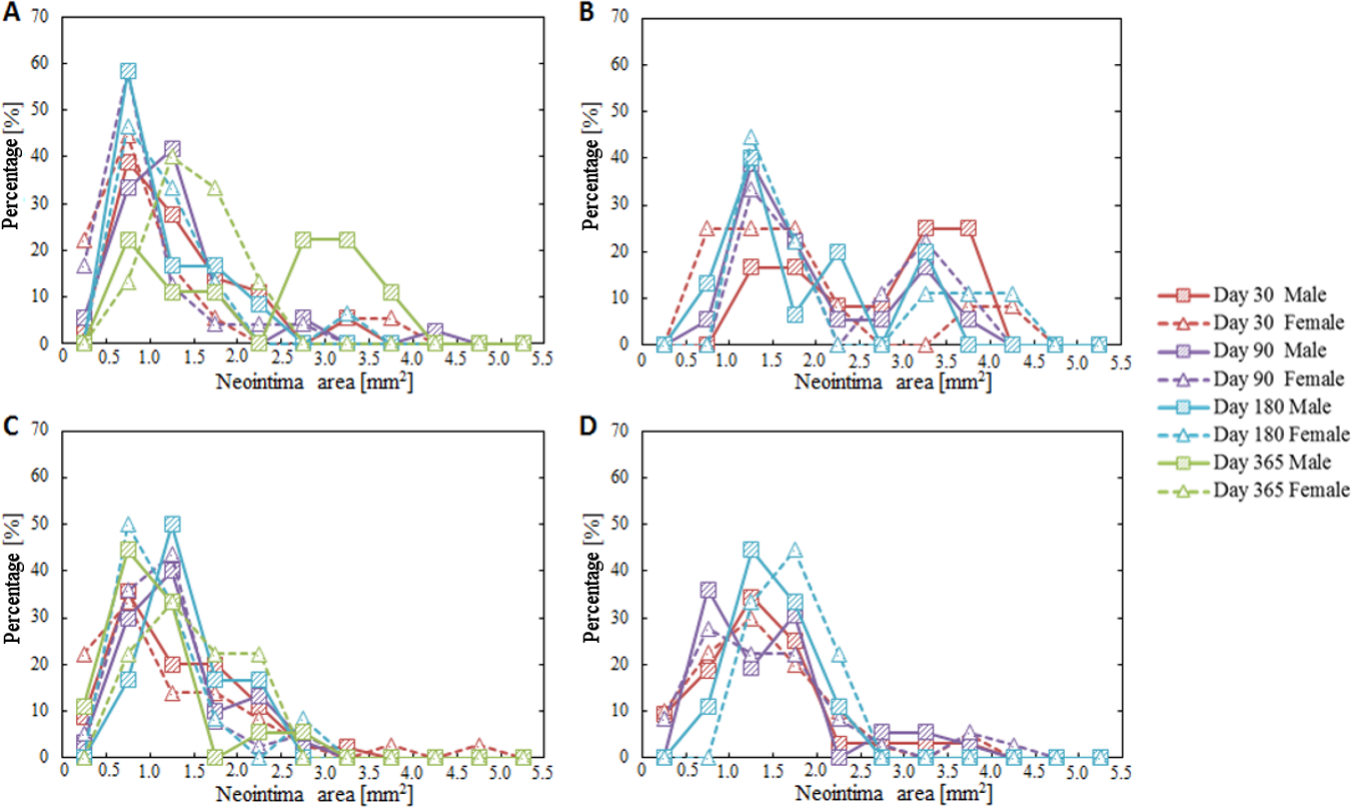
Distribution of neointima area. (A) Single BMS implantation, (B) Overlapped BMS implantation, (C) Single DES implantation, (D) Overlapped DES implantation. No significant differences between female and male groups were observed.

**Fig 3:**
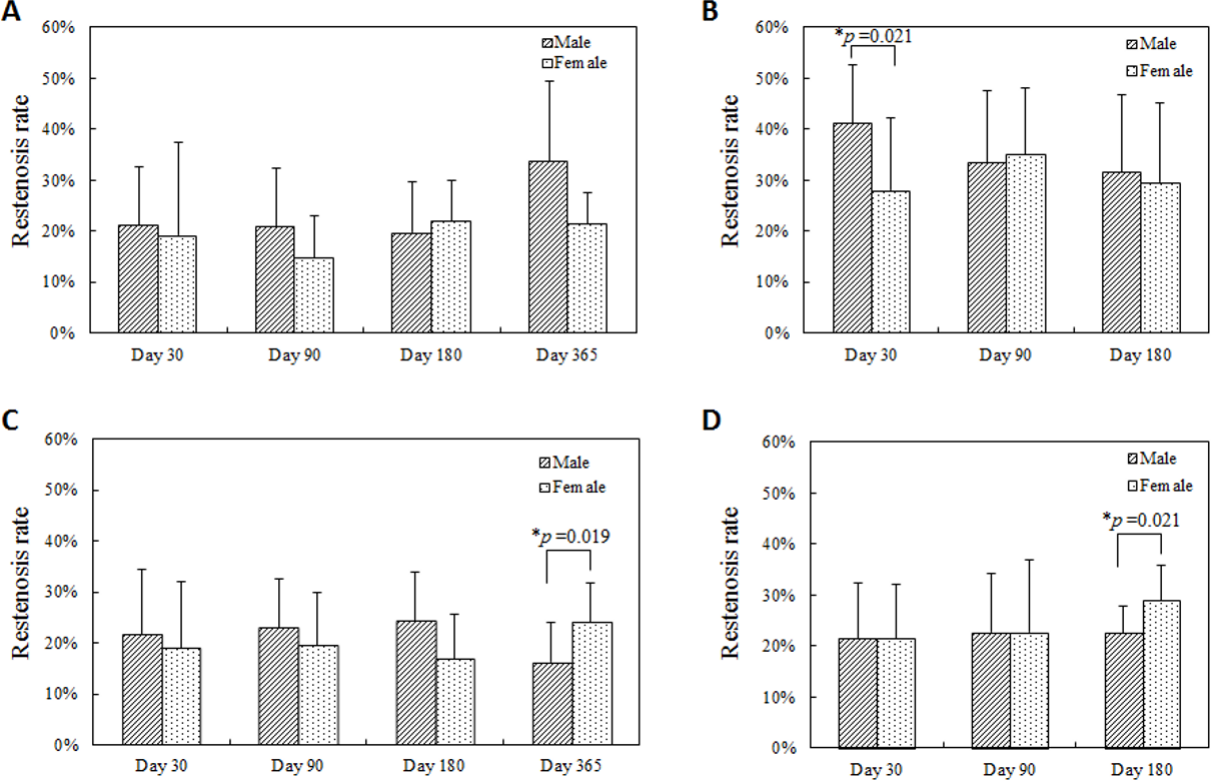
Luminal occlusion at each follow-up timing. (A) Single BMS implantation, (B) Overlapped BMS implantation, (C) Single DES implantation, (D) Overlapped DES implantation. Higher luminal occlusion with statistically significant difference were observed 30 days after overlapped BMS implantation in the male group (B), 365 days after single DES implantation in the female group (C), and 180 days after overlapped DES implantation (D).

**Fig 4:**
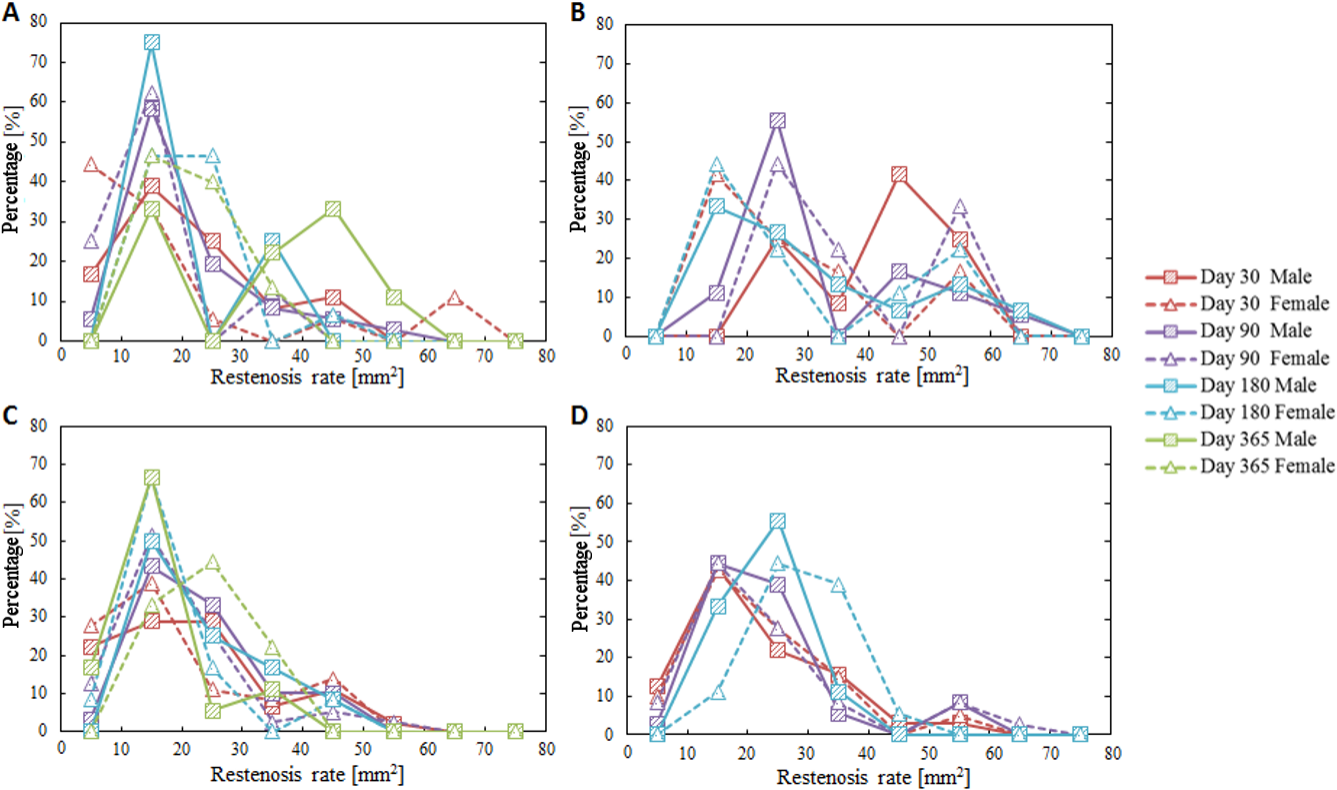
Distribution of luminal occlusion. (A) Single BMS implantation, (B) Overlapped BMS implantation, (C) Single DES implantation, (D) Overlapped DES implantation. No significant differences between female and male groups were observed.

Though mean neointima area did not reach statistically significant differences between female and male mini-swine under any procedure protocols at any time after implantation (Figs 1 and 2), there was a subtle difference in the timing of vascular recovery. Higher luminal occlusion with statistically significant differences was observed 30 days after overlapped BMS implantation in the male group, 365 days after single DES implantation in the female group, and 180 days after overlapped DES implantation in the female group (Fig 3). However, no significant difference between female and male groups was observed in the distribution of luminal occlusion at any follow-up time under any procedure protocols (Fig 4).

### Injury and inflammation

**Fig 5:**
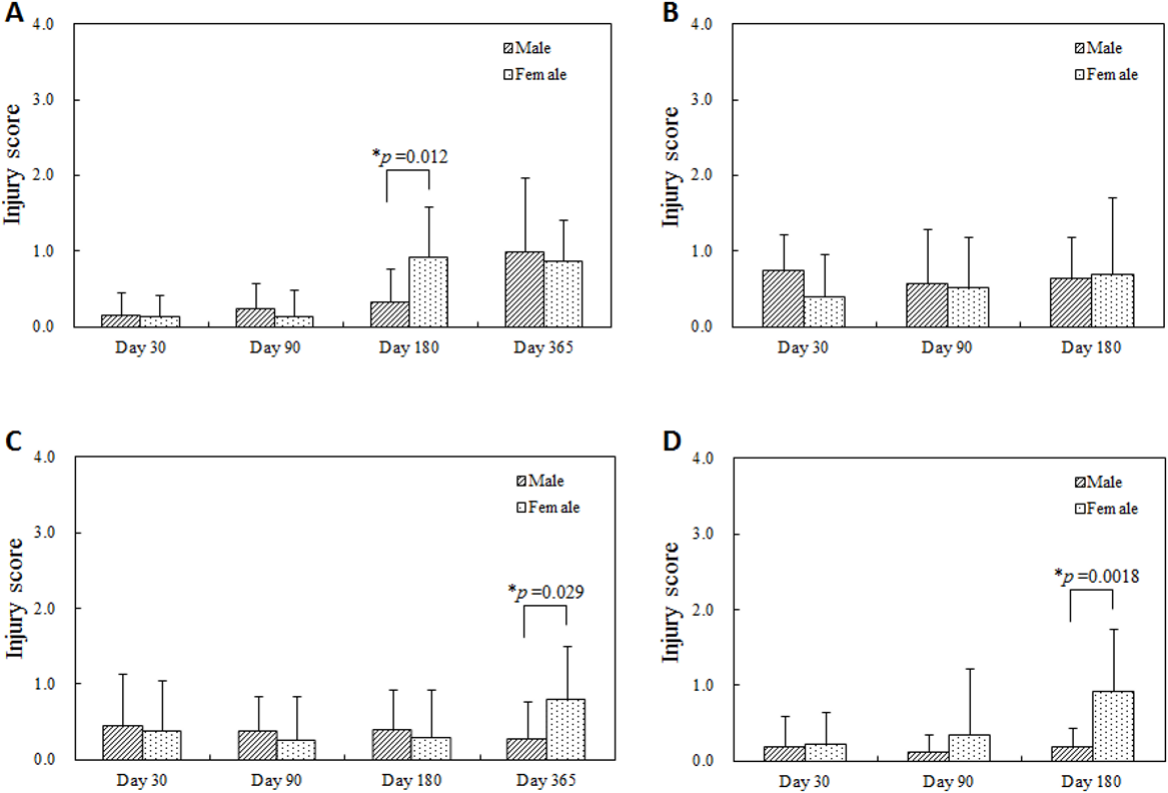
Injury score at each follow-up timing. (A) Single BMS implantation, (B) Overlapped BMS implantation, (C) Single DES implantation, (D) Overlapped DES implantation. Higher injury scores were observed in the female group 180 days after single BMS implantation (A), 365 days after single DES implantation (C), and 180 days after overlapped DES implantation (D).

**Fig 6:**
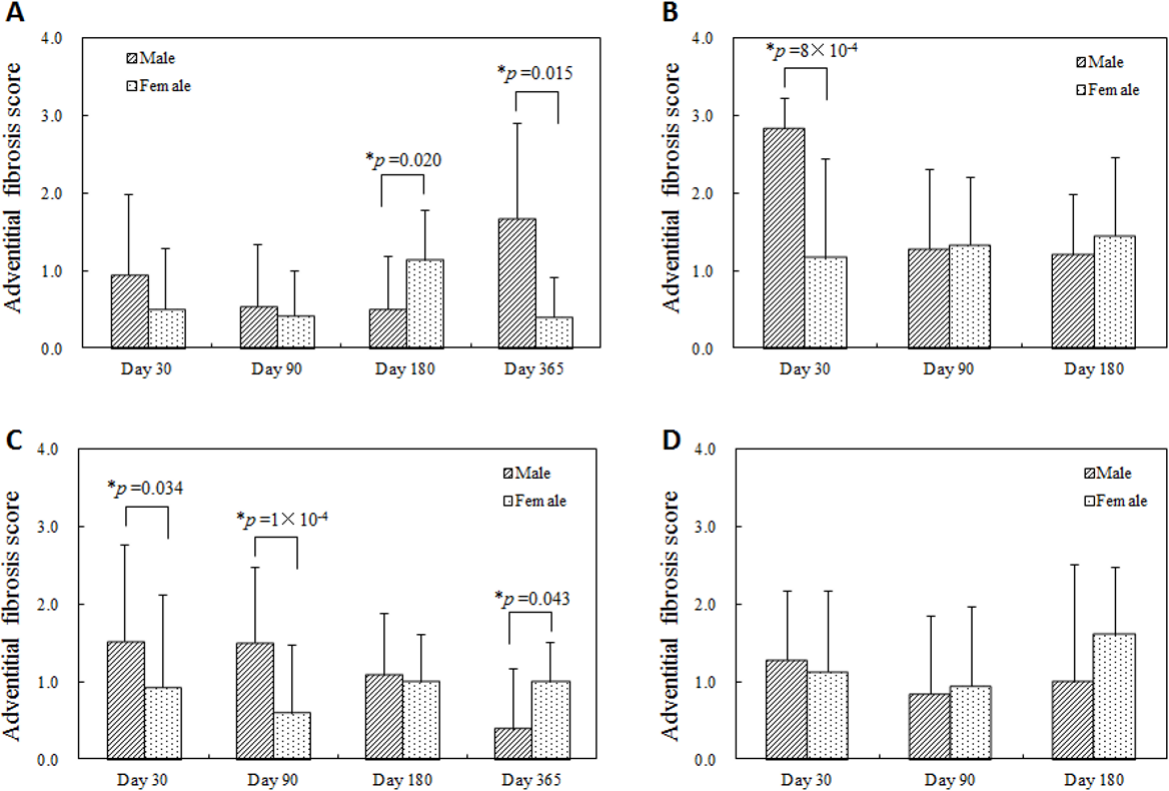
Adventitial fibrosis score at each follow-up timing. (A) Single BMS implantation, (B) Overlapped BMS implantation, (C) Single DES implantation, (D) Overlapped DES implantation. Higher adventitial fibrosis scores were observed 180 days after single BMS implantation in the female group (A), 365 days after single BMS implantation in the male group (A), 30 days after overlapped BMS implantation in the male group (B), 30 and 90 days after single DES implantation in the male group (C), and 365 days after single DES implantation in the female group (C).

**Fig 7:**
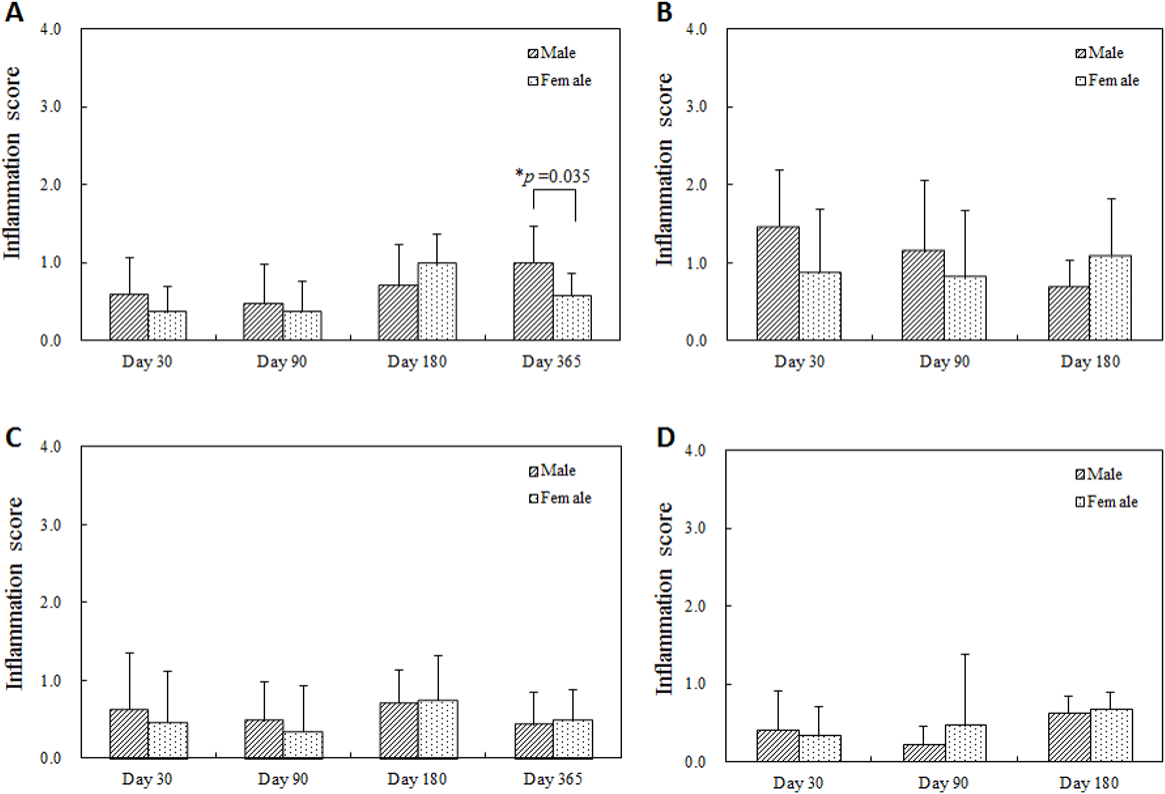
Inflammation score at each follow-up timing. (A) Single BMS implantation, (B) Overlapped BMS implantation, (C) Single DES implantation, (D) Overlapped DES implantation. A higher inflammation score with statistical significance was observed in the male group 365 days after single BMS implantation (A).

Similarly, though vascular injury was minimal (injury scores less than 1) for both sexes, injury was more statistically persistent and elevated at later times in the females; 180 days after single BMS implantation, 365 days after single DES implantation, and 180 days after overlapped DES implantation (Fig 5). Perhaps, as a consequence, there were higher adventitial fibrosis scores with statistical significance at these later times in the female group and earlier in the male group (Fig 6). Inflammation was limited and only higher in males 365 days after single BMS implantation, but no statistically significant differences were observed in other conditions (Fig 7). For all follow-up timings and for all procedures, neointimal maturation and endothelialization were complete.

### Neointimal fibrin score

**Fig 8:**
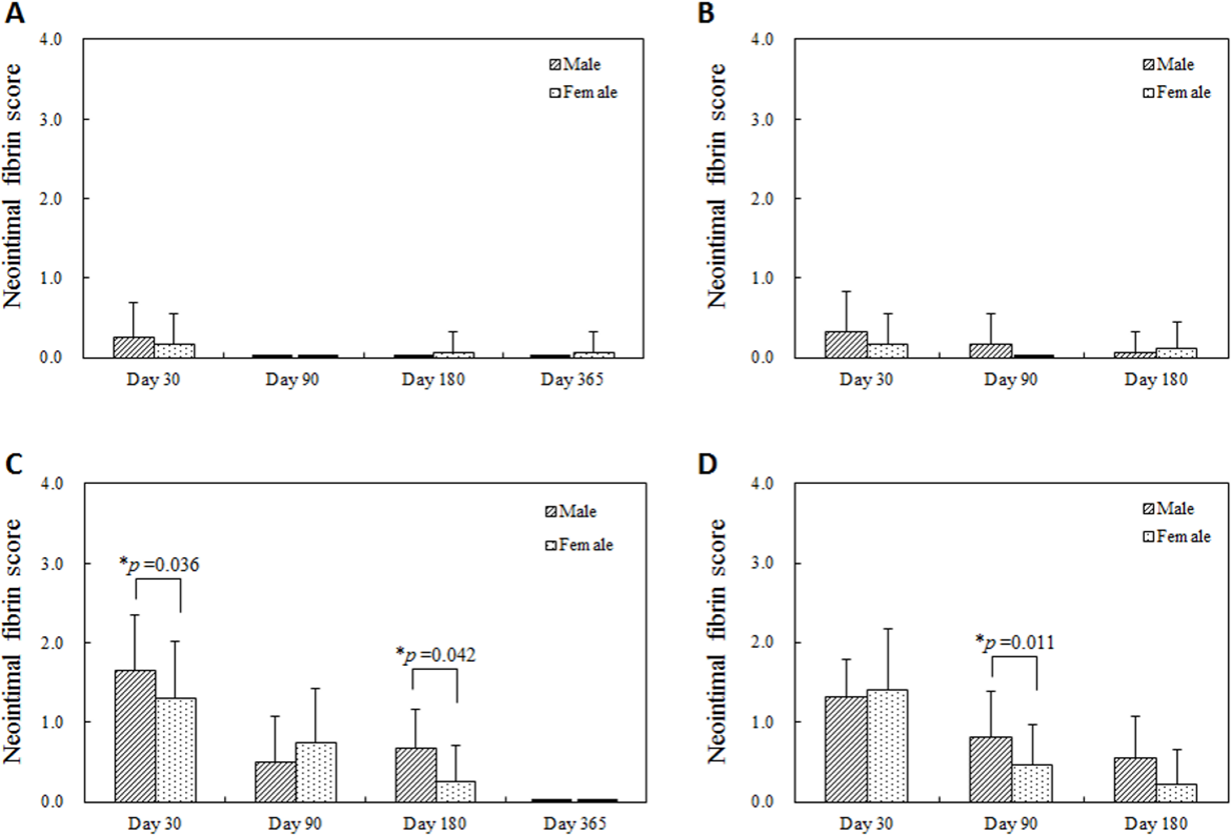
Neointimal fibrin score at each follow-up timing. (A) Single BMS implantation, (B) Overlapped BMS implantation, (C) Single DES implantation, (D) Overlapped DES implantation. Black lines in the male group at 90, 180, and 365 days after single BMS implantation and at 365 days after single DES implantation, and in the female group at 90 days after single BMS implantation, at 90 days after overlapped BMS implantation, and at 365 days after single DES implantation indicate that the neointimal fibrin scores were 0. A higher neointimal fibrin score was observed in male group 30 and 180 days after single DES implantation (C), and 90 days after overlapped DES implantation (D). The statistically significant decreases over time were observed in the female group after single and overlapped DES implantation (C, D).

Neointimal fibrin is a maker of drug delivery as it correlates with vascular fragility from local drug depots. Statistically higher neointimal fibrin scores were observed in male swine arteries with DES 30 and 180 days after single and 90 days after overlapped implantation (Fig 8). Fibrin scores decreased significantly more rapidly in the female swine as well.

## Discussions

Sex differences in vascular responses to catheter-based intervention have been sought with just cause. Several studies observed the sex-related difference in onset of cardiovascular events [8, 11], which has induced an on-going debate with regard to the role of sex hormones in cardiovascular disease [11, 45]. Women tend to bleed more during and after vascular procedures than men do and women are more prone to clotting due to higher baseline platelet activity than men [5]. However, the sex-related differences in outcomes of intervention have not been understood clinically because it is hard to balance sexes in the clinical trials. The National Institute of Health (NIH) has encouraged the scientific community to improve the balance in research [46], and all NIH grants must now document the consideration of sex as a biological variable. Given the control over conditions that are so inherently variable clinically like environment and tissue state and dimensions the preclinical domain is an excellent venue to examine sex-dependent issues [47]. Yet, few if any have looked at whether sex plays a role in the response to endovascular implantation in classic animal models. Coronary arteries of domestic crossbred or mini-swine, or rabbit iliac arteries are considered as the classic animal models for human vascular diseases [48], and FDA approval requires examination of devices, including BMS and DES, in swine models. Despite requiring that sex should be considered in research, we still have never formally examined whether animals should be balanced for sex or whether this issue is moot. To the best of our knowledge, this study attempted for the first time to reveal sex-related differences in outcomes of stent implantation in preclinical setting.

We pooled data from an extensive database of studies with stents and looked across a wide array of histomorphometric responses segregated by sex. While there were no major sex-related differences in neointima formation as the primary determinant of vascular repair in any of stent implantation procedures, there were statistically significant differences on a variety of parameters that might well emerge as important in specific instances. For example, injury and inflammation persisted for longer in the female swine and adventitial fibrosis and neointimal fibrin deposition were greater in in the males, which is consistent with the clinical trial results.

We need though to careful contextualize our findings since we seek not to recapitulate the human condition in the animal or even to answer whether vascular interventions are different in women and men but rather to understand if the biology in animals is sex-dependent. Accordingly let us first comment on what it means to be a male and female animal, second to understand if any confounding variables like arterial dimensions or device type could have biased the results, and then to understand the biologic consequences of variations in component parts of the repair pathways and final lesion formed.

### Sex in an animal

Animals are not humans and indeed animal models are used to investigate mechanisms and hypotheses not to predict responses in people. Animals in experimental model departs from the humans in clinical trials. Aside from the obvious age difference and environmental exposures, the notions of sex are different in experimental models and trial subjects. Almost all the animals used in this study are 4 months old and are often raised on carefully controlled chow in carefully modulated and monitored environments. There is no hypertension or diabetes mellitus unless introduced artificially, or there is no tobacco abuse or “unhealthy” lifestyle. More importantly for us, femaleness and maleness have different insinuations. Male animals in experimental systems are most often neutered and female swine most often pre-menarchal placing animals on the different extreme of the natural spectrum of sex hormonal exposure than older human subjects. Although the mini-swine reproductive system is similar to that of human, mini-swine are sexually matured at 5–6 months old, while these animals are usually 4 months old at the initiation of preclinical studies. In addition, these studies mostly follow up to 12 months, when sexual maturation begins. As studies go longer and longer, especially when we begin to examine stents with erodible coatings or backbones, the sexual maturation may overlap with follow-up and we may understand the influence of sex on the outcomes. Thus, our data are valuable to scientists especially in an era where federal mandates to consider sex as a biological variable are ubiquitous but we are careful not to claim association with the human condition.

### Confounding variables in preclinical trials

Preclinical experiments are carefully mandated and especially as all the experiments represented here were performed not only in an AAALAC-accredited facility but under GLP guidance as well. Sex-related difference in clinical outcomes of stent implantation may well have arisen from anatomic differences between women and men as arterial dimensions are smaller in women than in men [3–5]. This was not the case in our pooled animal data. There was no statistically significant difference in baseline arterial diameter or post-implant diameter between male and female swine in 16 out of 18 different device type and follow-up timing group. In the other 2 groups, in which statistically significant difference was observed, the diameters were minimally greater in the female not the male swine (Table 2). This lack of difference in dimensions is of no surprise given that preclinical protocols careful dictate ranges of diameters and given that dimensions are critical in the animal anatomic identity is essential. Such matching strengthens the arguments of these findings.

Different hemodynamic patterns could potentially influence the outcomes of stent implantation. In the pooled data, the stents were implanted in all three coronary arteries and we and others have shown differential physical forces and hemodynamic patterns on the different coronary arteries [49]. Although statistical analysis could not be performed by subcategorizing our analysis by each coronary artery due to the limited sample size, no difference was observed in neointima area, restenosis rate, or any histomorphological parameters among three coronary arteries. Single-usage or overlapped-usage also create different hemodynamic pattern and net effects were identical to single stenting.

### Component pathways and cumulative biologic effect

One important element from this work is that overall cumulative effects on an artery like ultimate neointimal hyperplasia and thrombosis are no different in female and male swine though different pathways and elements of the response to injury do show differential effects. This is an important finding and not contradictory. Neointima or clot formation are the end result of a multitude of processes and are subject to the full range of biologic, biochemical, physical and hemodynamic effects. The metrics integrate all of the net biologic effects that confront the artery, and it is impossible to predict the end result from isolated component parts. This may well explain why effects like fibrin deposition cannot predict overall thrombosis, or inflammation and proliferation cannot predict ultimate neointimal mass. Cumulative effects integrate many events such as arterial geometry and flow as well as physiology and biology and it is essential that these two sets of effects are reported independently and precisely.

That differences in specific biological pathways after stent implantation bare metal and drug-eluting, with regard to sex is important in helping investigators plan and justify future studies. This is especially the case as subtle sex-related differences may be of increasing importance as interventional devices meld novel materials that erode and innovations in drug delivery. Erodible materials may act differently if inflammation has a different temporal sequence with sex, and drug distribution after balloon or stent delivery might be different if the fibrin clearance speaks to different modes of pharmacokinetics in male and female swine.

## Conclusions

In this study, we used a pooled data of catheter-based interventions in more than 200 coronary arteries of Yucatan mini-swine to understand sex-related influence on vascular repair after stent implantation. Animals were split almost equally between males and females and both bare metal and drug eluting stents were considered in single and overlapped configurations. While several clinical studies observed sex-related differences in cardiovascular events, our pooled data showed no sex-related differences in the major bulk properties of thrombosis, neointima formation or luminal occlusion at all times in follow-up from 1 month to 1 year after stent implantation. Statistically significant differences between male and female swine were observed in histomorphological parameters directed at resolution of fibrin deposition after drug delivery and degree of inflammation. These issues speak to the mandate to consider sex as a biological variable in animal experiments and will likely become important when we consider complex convergence technologies such as erodible materials where the dynamics of vascular repair after initial events determine late effects.

## Supporting information

S1 Appendix Detailed description of entire procedure. (PDF)

S2 Appendix Histomorphological assessment and its robustness. (PDF)

S3 Appendix ARRIVE guidelines checklist. (PDF)

S1 Table All the anatomical and histomorphological data. (XLSX)
