# Supplementary material for: Sex differences in the outcomes of stent implantation in mini-swine model
Source: PLoS One. 2018 Jan 29;13(1):e0192004. doi: 10.1371/journal.pone.0192004 (PMC5788368; doi:10.1371/journal.pone.0192004)
Supplement: S1 Appendix — (PDF) [file pone.0192004.s001.pdf]

S1 Appendix – Detail Description of Procedure

To prevent or reduce the occurrence of thrombotic events, aspirin (650 mg, *per os* [PO]) and clopidogrel (300 mg, PO) were administered on Day -1, Day -2, or Day -3. Aspirin (81 mg, PO) and clopidogrel (75 mg, PO) were generally administered daily thereafter until euthanized. Animals were not offered their daily food ration before surgical induction to decrease the chances of peri-operative regurgitation. Water was not restricted.

Buprenorphine (0.01 mg/kg, intramuscularly [IM]) was administered at the time of anesthetic induction. Telazol® (4–6 mg/kg, IM) was administered as a pre-anesthetic. Isoflurane anesthesia was administered to effect via mask/nosecone until the animal was in a plane of anesthesia that facilitated endotracheal intubation. Once sufficiently anesthetized, the animal was intubated and maintained with isoflurane inhalant anesthetic to effect for the remainder of the interventional procedure. Preoperative nifedipine (10 mg/animal placed sublingually) was administered. An intravenous (IV) catheter was placed in a peripheral vein for administration of supportive IV fluids. An ophthalmic lubricant was applied to the eyes. Animals were prepared for surgery/aseptic procedures using accepted veterinary care standards.

The animal was moved to the surgical suite and placed in dorsal recumbency. Supportive fluids were administered and the fluid type and volume administered was documented in the animal's record. Warm water heating pads and/or other warming devices were used to help maintain adequate body temperature while under anesthesia. Electrocardiogram, heart rate, respiratory rate, SpO_2, temperature and blood pressure (direct or indirect) were monitored and documented at regular intervals during the surgical procedure.

After induction of anesthesia, access was made in a femoral artery via cutdown. An arterial sheath was introduced and advanced into the artery. Heparin (150 U/kg, IV or intra-arterial [IA]) was administered on Day 0 (after placement of the introducer sheath) to prolong ACT to a target of approximately 275 seconds. Arterial blood samples (< 0.5 mL each) were collected within 5-10 minutes

of heparin administration. When ACT monitoring indicated a value that was less than the target, a second test was run with a new blood sample to confirm accuracy and to avoid over anti-coagulation.

Under fluoroscopic guidance, a guide catheter was advanced through the sheath into the ascending aorta and to the coronary arteries. Nitroglycerin (200 μg, IA) was administered into the coronary arteries before the angiograms. Angiographic images of the vessels were obtained with contrast media to identify the proper location for the deployment site.

Each animal received up to 3 stents (varying stent groups); one in the left circumflex (LCX), left anterior descending (LAD), and/or right coronary (RCA) arteries, and/or branches thereof. The stents were expanded during placement to a target balloon:artery ratio of 1.1:1-1.2:1. Stents were introduced into the coronary arteries by advancing the stent delivery system through the guide catheter and over the guide wire to the deployment site within the coronary artery. Confirmation of balloon:artery ratio was made when the angiographic images were quantitatively assessed. After the target balloon:artery ratio was achieved, vacuum was applied to the inflation device in order to deflate the balloon. Complete balloon deflation was verified with fluoroscopy. While maintaining guide wire position, the delivery system was then slowly removed. Any resistance during delivery or removal of the stent delivery system was noted. Contrast injections were used to determine device patency and additional acute deployment characteristics.

At the conclusion of the procedure, the accessed vessel(s) were ligated or repaired. The surgical incisions were closed with appropriate suture techniques and materials. Any animal placed on mechanical ventilation was gradually weaned off ventilatory support and continuously monitored and supported with manual ventilation (if needed) until spontaneous respirations resumed. Animals were continuously monitored until endotracheal extubation occurred. The animals were extubated and post-operative care procedures were performed in accordance with accepted veterinary standards. Animals were monitored until they recovered fully from anesthesia. Buprenorphine (0.01 mg/kg, IM or SC) was administered at least once every 12 hours to provide analgesic coverage through at least the first 24 post-

operative hours and then as needed based on clinical assessment. Daily food ration was provided upon recovery from anesthesia.

At each designated endpoint, animals were given Telazol (4–6 mg/kg, IM) and anesthetized via isoflurane inhalant as described previously. After prenecropsy angiography, each animal was euthanized via an overdose of potassium chloride solution (IV) in accordance with accepted American Veterinary Medical Association (AVMA) guidelines.
