# Supplementary material for: Sex differences in the outcomes of stent implantation in mini-swine model
Source: PLoS One. 2018 Jan 29;13(1):e0192004. doi: 10.1371/journal.pone.0192004 (PMC5788368; doi:10.1371/journal.pone.0192004)
Supplement: S2 Appendix — (PDF) [file pone.0192004.s002.pdf]

S2 Appendix – Histomorphological assessment and its robustness

Quantitative morphometric analysis was performed on the histological sections from each stented artery. For each histological section, the parameters were directly measured using standard light microscopy and computer-assisted image measurement systems. From these direct measurements, all other histomorphometric parameters were calculated.

Stented Vessel Histomorphometry				
Directly Measured Metrics	Parameter		Abbreviation	Unit
	Lumen Area		L_a	mm^2
	Internal Elastic Layer (IEL) Bounded Area		IEL_a	mm^2
	Stent Area		S_a	mm^2
	Artery Area / External Elastic Layer (EEL) Bounded Area		A_a	mm^2
Calculated Metrics	Parameter	Calculation	Abbreviation	Unit
	Area Measurements			
	Neointimal Area	$IEL_a – L_a$	N_a	mm^2
	Medial Area	$A_a – IEL_a$	M_a	mm^2
	Length Measurements			
	Lumen Diameter	$2 \times \sqrt{(L_a/\pi)}$	L_d	mm
	IEL Diameter	$2 \times \sqrt{(IEL_a/\pi)}$	IEL_d	mm
	Ratios			
	Lumen Area / Artery Area	L_a/A_a	$L_a:A_a$	NA*
	Restenosis Parameters			
	Percent Area Stenosis	$N_a/(IEL_a) \times 100\%$	AS	%
	Neointimal Thickness	$(IEL_d – L_d)/2$	N_{mm}	mm
*NA = Not Applicable				

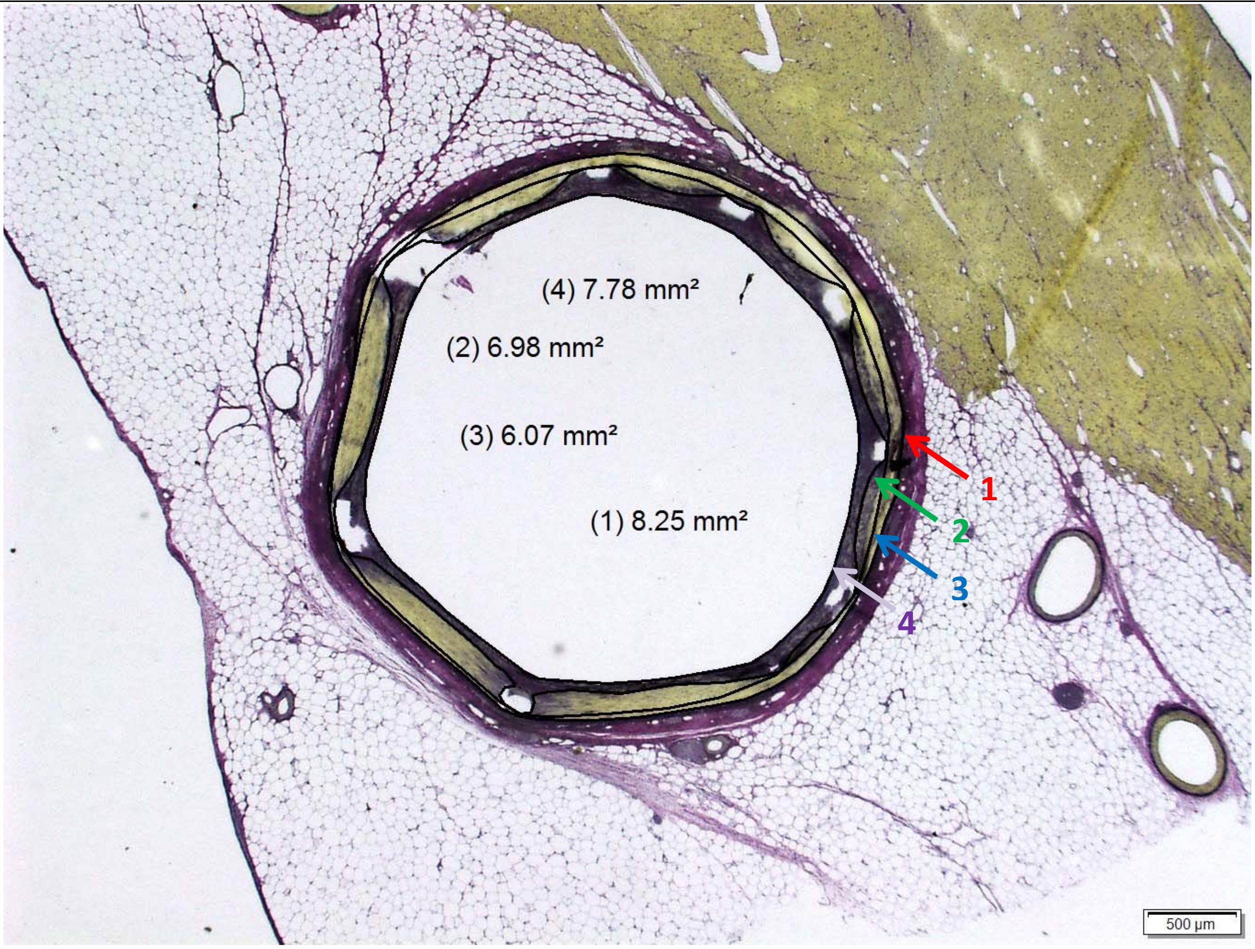

Figure S1. BMS Stent Middle Plane 1.25x VER

Each image contains four measurements taken in consecutive order. The following sequence represents their correlating biological structure: (1) Area encompassed by the External Elastic Lamina (EEL) [mm^2], (2) Area encompassed by the Internal Elastic Lamina (IEL) [mm^2], (3) Area encompassed by the luminal surface [mm^2], (4) Area encompassed by the stent perimeter [mm^2].

Light microscopy was used in histomorphological scoring of parameters reflecting the host response/repair process to the treatment. Inflammation and injury scores were evaluated for each strut basis, while all other parameters were scored per slide.

**Stented Vascular Histomorphology Scoring Matrix**	
Score	**Injury (0 – 3)**
0	No injury, internal elastic lamina (IEL) intact.
1	Disruption of IEL.
2	Disruption of tunica media.
3	Disruption of the external elastic lamina (EEL) / tunica adventitia.
Score	**Inflammation (0 – 3)**
0	Absent, no cells present.
1	Fewer than ~ 20 cells associated with stent strut.
2	Greater than ~20 cells associated with stent strut, with or without tissue effacement and little to no impact on tissue function.
3	Greater than 20 cells associated with stent strut, with effacement of adjacent vascular tissue and adverse impact on tissue function.
Score	**Endothelialization (0 – 4)**
0	Absent, no endothelial cell coverage.
1	< 25% of luminal surface covered by endothelial cells.
2	25% to 75% of luminal surface covered by endothelial cells.
3	> 75% of luminal surface covered by endothelial cells.
4	100% of luminal surface covered by endothelial cells, confluent.
Score	**Neointimal Fibrin (0 – 3)**
0	Absent.
1	Minimal, infrequent, light, focal to multifocal, spotting of fibrin, generally limited to peri-strut neointima.
2	Mild to moderate, multifocal to focally extensive deposition of fibrin with tendency to coalesce and form solid accumulations which extend beyond peri-strut neointima and/or to an adjacent strut.
3	Marked, widespread to focally extensive coalescing accumulations of fibrin which effaces normal tissue architecture and spans between/beyond 2 or more struts.
Score	**Neointimal Maturation (0 – 3)**
0	Absent.
1	Immature, predominantly fibrino-vascular tissue.
2	Transitional, predominantly organizing smooth muscle.
3	Mature, generalized, organized smooth muscle.
Score	**Adventitial Fibrosis (0 – 3)**
0	Absent.
1	Minimal presence of fibrous tissue.
2	Notable fibrous tissue in 25 – 50% of artery circumference.
3	Notable fibrous tissue in > 50% of artery circumference.
Score	**Pertinent Microscopic Observations**
0	No observable change
1	A nearly imperceptible (minimal) feature/change in the tissue
2	An easily identifiable or notable (mild / moderate) feature/change in the tissue
3	Prominent to overwhelming (marked / severe) feature/change in the tissue

CBSET, Inc. has over ten years' experience performing a GLP-compliant vascular morphometric analysis. Consistency and accuracy in morphologic measurements are maintained via a series of SOP-driven controls along the procedures. Some minor variation in tissue sectioning and tissue measurements is unavoidable, but tissue trimming and sectioning are performed to maximize transverse sectioning and minimize any anatomic variation and tissue artifacts. Identification of vascular structures and subsequent morphometric analysis is performed by trained individuals, with final oversight of ACVP-certified veterinary pathologists.

Impact of inflammation and other parameters on tissue function is likewise interpreted by veterinary pathologists experienced with a wide variety of vascular interventions, vascular structures, tissue responses, timepoints and outcomes. The scoring paradigm utilized in the relevant studies, on a 0-3 grade scale, was designed to both semiquanitatively assess inflammatory cell infiltrates and their likely significance. In the relevant studies, Grade 0 indicates no inflammatory cells present, while Grade 1 indicates a small number of cells (<~ 20) immediately associated with a stent strut as is typical of a uncomplicated presence of a device. Grade 2 indicates more numerous (>~ 20 cells) surrounding a stent strut, possibly associated with localized obscuring of tissue architecture and interpretation of little to no impact on tissue function. Grade 3 inflammation includes a similar number of inflammatory cells with effacement of adjacent vascular tissue and presumption of adverse impact on tissue function. The significance of changes is therefore based on degree of changes in tissue and application of established guidelines and experiential knowledge of outcomes across *in vivo* studies.
